# Gamma band activity associated with BCI performance: simultaneous MEG/EEG study

**DOI:** 10.3389/fnhum.2013.00848

**Published:** 2013-12-06

**Authors:** Minkyu Ahn, Sangtae Ahn, Jun H. Hong, Hohyun Cho, Kiwoong Kim, Bong S. Kim, Jin W. Chang, Sung C. Jun

**Affiliations:** ^1^School of Information and Communications, Gwangju Institute of Science and TechnologyGwangju, South Korea; ^2^Korea Research Institute of Standards and ScienceDaejeon, South Korea; ^3^Department of Neurosurgery, Brain Research Institute, Yonsei University College of MedicineSeoul, South Korea; ^4^Wadsworth Center, New York State Health Department, AlbanyNY, USA

**Keywords:** BCI-illiteracy, motor imagery BCI, performance prediction, gamma activity, MEG, EEG

## Abstract

While brain computer interface (BCI) can be employed with patients and healthy subjects, there are problems that must be resolved before BCI can be useful to the public. In the most popular motor imagery (MI) BCI system, a significant number of target users (called “BCI-Illiterates”) cannot modulate their neuronal signals sufficiently to use the BCI system. This causes performance variability among subjects and even among sessions within a subject. The mechanism of such BCI-Illiteracy and possible solutions still remain to be determined. Gamma oscillation is known to be involved in various fundamental brain functions, and may play a role in MI. In this study, we investigated the association of gamma activity with MI performance among subjects. Ten simultaneous MEG/EEG experiments were conducted; MI performance for each was estimated by EEG data, and the gamma activity associated with BCI performance was investigated with MEG data. Our results showed that gamma activity had a high positive correlation with MI performance in the prefrontal area. This trend was also found across sessions within one subject. In conclusion, gamma rhythms generated in the prefrontal area appear to play a critical role in BCI performance.

## INTRODUCTION

Over the past several decades, considerable attention has been paid to the subject of brain computer interface (BCI) technology (Wolpaw et al., 2002), as it is an attractive notion that BCI can translate a user’s intention or mental state through brain waves. From the early days of this research until now, and especially in recent decades, BCI has been improved and its accuracy has been much enhanced with the help of machine learning algorithms. For control use, motor imagery (MI)-based BCI has been one of the most popular designs, such as P300 and steady state visual evoked potential (SSVEP) BCIs (Bashashati et al., 2007; Guger et al., 2011). Pfurtscheller and Lopes da Silva (1999) observed that mu-rhythms over sensorimotor areas involved with motor function are attenuated notably when a person imagines body part movement. Brain signal patterns associated with this phenomenon are extracted commonly for the purpose of controlling the system.

The investigations and development of this technology have been conducted in non-invasive (Wolpaw et al., 2002; Blankertz et al., 2006, 2008a; Jerbi et al., 2011; Ortner et al., 2012) as well as invasive systems (Leuthardt et al., 2004; Jerbi et al., 2009). Recent studies have reported success in decoding the direction of movement (Mehring et al., 2004; Rickert et al., 2005; Waldert et al., 2008, 2009; Ball et al., 2009), target (Hammon et al., 2008; Ubeda et al., 2013), velocity (Bradberry et al., 2009; Lv et al., 2010; Ofner and Müller-Putz, 2012; Robinson et al., 2013), trajectory (Schalk et al., 2007), grasp type (Pistohl et al., 2012) and real-time detection of visuo-spatial working memory (Hamamé et al., 2012).

It is expected that these successes may soon be applied to BCI and allow public marketing. However, obstacles that need to be resolved still exist, even in accurate BCI systems; a crucial hurdle is that BCI performance varies significantly across and even within users. Reportedly, approximately 20–30% of target users do not generate controllable brain signals (extractable and classifiable by existing techniques) in MI (Guger et al., 2003; Blankertz et al., 2008b; Ahn et al., 2013). Such problems exist commonly in other current BCI systems and must be overcome before BCI technology can advance further. Even though one shows good decoding accuracy during the calibration phase, it may yield poor performance in the online phase; alternatively, although one may be able to control a BCI system at one time, a loss of control may occur at another time. This indicates that performance variation is observed not only across people, but also at different times within subjects. Therefore, understanding why this problem occurs and investigation of its causes/correlates are important in making BCI a usable interface.

In recent studies, efforts to find correlates with BCI performance have yielded interesting outcomes. Blankertz et al. (2010) reported the importance of idling alpha activity in the motor cortex, which is associated implicitly with a potential decrease of power below baseline. They observed that subjects with high alpha levels during the resting state are likely to have great potential to yield larger power decreases, which can be used as a main feature for MI BCI. Further, Ahn et al. (2013) found that high alpha and low theta is a typical pattern among those who perform MI BCI well. These findings are highly beneficial in establishing applicable BCI systems in both healthy and ill users. It is possible to pre-screen users of the BCI system, and those who demonstrate poor BCI performance may be trained through biofeedback before actual BCI use (Hwang et al., 2009). Even users themselves may decide without difficulty to choose other interfaces or BCI control paradigms that are better suited for them (Volosyak et al., 2010).

Another noteworthy factor is psychological state. It has been reported that motivation (Leeb et al., 2007; Hammer et al., 2012) and mindfulness (Mahmoudi and Erfanian, 2006; Lakey et al., 2011) influence BCI performance. In an intensive study with 83 subjects, the ability for visuo-motor coordination and concentration on a task was revealed to have a significant positive correlation with classification accuracy of MI (Hammer et al., 2012). Nijboer et al. (2010) concluded that motivational factors may be related to performance in patients with amyotrophic lateral sclerosis (ALS). Fear of the BCI system has also been suggested as a factor that degrades performance (Burde and Blankertz, 2006; Nijboer et al., 2010; Kleih et al., 2013; Witte et al., 2013). Burde and Blankertz (2006) reported that highly confident subjects are likely to show better control in MI BCI.

Performance variability is observed not only between, but also within users. Usually, BCI performance is estimated by means of hit-trials in online cases and average accuracy through cross-validation in offline analysis. However, subjects may yield different performance over runs, even within the same experiment. Performance may fluctuate considerably when a user is tested over a period of days, as environment and mental states vary over time. Moreover, this variability may occur even within seconds across trials. Grosse-Wentrup et al. (2011) tried to explain the trial-wise variability in relation to the gamma (55–85 Hz) rhythm. They reported that the causal effect of gamma on sensory motor rhythm (SMR) was induced through the framework of causal influence (Pearl, 2000; Spirtes et al., 2001). The empirical results of Grosse-Wentrup et al. (2011) revealed that gamma activity in the fronto-parietal network played a critical role in the MI process. Frontal gamma may affect MI ability because high frequency oscillations reflect attention and cognitive processes (Uhlhaas et al., 2009). Therefore, it has been inferred that the level of a user’s concentration has some effect on the process of imagining body movement. Conversely, increases in gamma power in corresponding areas during MI have also been reported (Pfurtscheller and Lopes da Silva, 1999). In an invasive study, further support for this gamma increase was obtained in the electrocorticogram (ECoG) work of Aoki et al. (1999). They found a power decrease in the 11–20 Hz range and an increase in the 31–60 Hz range in the forearm sensorimotor cortex during performance of visuo-motor tasks. As the hypothesis that gamma reflects functions in a specific brain area with highly precise synchronization in local networks (Fries et al., 2007; Womelsdorf et al., 2007), Halder et al. (2011) found in an fMRI study that the number of activated voxels in the supplementary motor area (SMA) was larger for users with relatively higher MI ability. Therefore, it is evident that gamma increases on SMA reflect a larger ensemble of neurons for certain types of processing (Jensen et al., 2007). In a trial-wise manner, Grosse-Wentrup et al. (2011) also reported a weak negative correlation between centro-parietal gamma oscillation and trial-wise SMR quality scores, which are the magnitude of output from a classifier, such as a support vector machine (SVM). This indicates that the brain state in which there are relatively low levels of gamma in the MI-related brain area during the resting state may facilitate the MI process because a high resting alpha rhythm is essential for good performance of MI BCI.

Summarizing these studies, two types of gamma originating from different brain areas seem to be important in MI:

• Frontal gamma, which influences MI indirectly
• Directly linked gamma, which increases in the centro-parietal area during MI

However, it remains unclear whether these gamma activities influence MI performance across or within subjects, or perhaps both. The influence of high frequencies (>30 Hz) above beta on MI performance has rarely been studied, although high frequency information is being investigated actively (Worrell et al., 2012). In particular, individual differences in gamma across subjects have never been investigated, and only one EEG study (Grosse-Wentrup et al., 2011) on trial-wise variability has been reported, although there have been some studies (Muthukumaraswamy et al., 2009, 2010) of visual gamma powers. In high frequency analyses, EEG has yielded reasonable results in some studies (Darvas et al., 2010; Grosse-Wentrup and Schölkopf, 2012); however, it is understood that EEG has low spatial resolution and volume conduction problems, especially in source reconstruction. For in-depth analysis of gamma activity, magnetoencephalography (MEG) is another choice; it has comparatively good temporal and relatively higher spatial resolution than EEG. For this reason, MEG facilitates the investigation of gamma, and thus has been introduced for MEG-based BCI (Lal et al., 2005; Mellinger et al., 2007; Buch et al., 2008; Battapady et al., 2009; Bianchi et al., 2010; Sudre et al., 2011; Zhang et al., 2011) and related work (Battapady et al., 2009; Bradberry et al., 2009; Ko and Jun, 2010; Wang et al., 2010; Hong and Jun, 2012; Chowdhury et al., 2013; Hong et al., 2013).

The purpose of this study was to identify individual differences in gamma activity and the extent of their influence on MI performance across subjects. For this purpose, a total of ten simultaneous MEG and EEG datasets were recorded from ten subjects. First, the MI classification accuracy of each subject was evaluated by EEG and the resting state of MEG was used for correlation analysis between gamma activity and BCI performance. Simultaneous MEG/EEG acquisition and methods are explained in Section “Materials and Methods.” Results are presented in Section “Results.” Finally, further interpretations and possible applications are discussed in Section “Discussion.”

## MATERIALS AND METHODS

### SUBJECTS

Ten subjects (ages: 25.3 ± 2.0 years old; 8 males, 2 females) participated in this study. The experiment was approved by the Institutional Review Board of Gwangju Institute of Science and Technology. All subjects were informed of the experimental process and purpose, and written consent letters were collected from them before the experiment.

### SIMULTANEOUS MEG/EEG DATA ACQUISITION

All experiments were conducted with MEG in a magnetically and electrically shielded room developed by Korea Research Institute of Standards and Science in South Korea (152 channels axial gradiometer, sampling rate: 1 kHz or 512 Hz, notch filtering at 60 Hz and bandpass filtering with 0.1–100 Hz), Biosemi EEG (19 channels electrodes, sampling rate: 512 Hz) and Brain Products EEG (19 channels, sampling rate: 500 Hz, notch filtering at 60 Hz) systems. EEG electrodes were attached to the entire scalp (Fp1, Fp2, F3, F4, C3, C4, P3, P4, O1, O2, F7, F8, T3, T4, T5, T6, Fz, Cz, Pz) according to the 10–20 international system. For three subjects (H, I and J), MEG/EEG was digitized at 1 kHz and down-sampled to 500 Hz using the Brain Products EEG system. Signals were recorded in two different states, the resting state, which is recorded at the beginning of the experiment, and the mental state while performing MI. Here, MEG and EEG were also recorded simultaneously, but EEG was used only to estimate the performance of MI BCI. In general, EEG has been used most often for BCI due to its portability and low cost; thus, BCI performance estimated by EEG alone is more realistic than that estimated by MEG alone or both MEG/EEG.

### RESTING STATE

Subjects were seated in a comfortable armchair and instructions were projected onto a screen approximately 80 cm away. The resting state signal was acquired before the MI task. This resting state acquisition lasted 60 s, while the subjects did nothing but let their minds wander with eyes open.

### MI TASK

**FIGURE 1:**
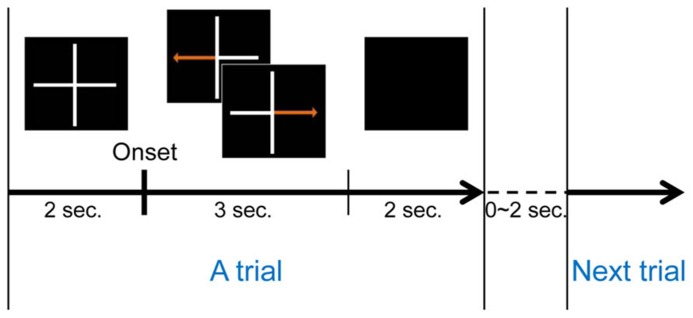
**One trial of MI task**.

A conventional left and right hand imagery movement task was introduced to estimate each subject’s MI ability. Each subject conducted three runs during this task. One run consisted of twenty trials for each class (left or right hand movement imagination), for a total of forty trials. A trial began with a gray fixation cross on a black background. Subjects were asked to move their eyes as little as possible during each trial. Either a left or right arrow appeared on the gray fixation cross after 2 s of preparation. The MI phase began with the appearance of a randomly selected directional arrow, and subjects were instructed to imagine their hand movement, such as making a fist with the left or right hand according to the arrow until it disappeared. The MI phase lasted 3 s for each trial. Afterward, the screen went blank and subjects were allowed to relax for 2 s. A randomized interval of 0–2 s was allocated between consecutive (randomly chosen right or left) trials to avoid subjects’ adaptation. **Figure 1** illustrates one trial of this MI experiment. A total of 120 trials were collected during the experiments.

### CLASSIFICATION ACCURACY FROM EEG

The MI trials acquired were used to quantify the subjects’ MI performance. As in previous studies (Ahn et al., 2012), each signal was bandpass-filtered with 8–30 Hz to include alpha and beta rhythms, as these bands are well known to contain very informative features that classify two different MI conditions (Pfurtscheller and Lopes da Silva, 1999; Ahn et al., 2012). Next, a temporally moving window with a window size of 2 s and sliding step of 100 ms were applied in order to calculate time variable classification accuracy from onset. The classification accuracy in each window was obtained through cross validation using 120 iterations, as follows: trials were separated into 10 groups containing an equal number of trials; for each iteration, 10 groups were divided randomly into 7 (training data) and 3 groups (testing data), respectively. The number of cases required to choose 7 out of 10 groups is 120; for each case, a classifier was constructed from the training data, and testing data were evaluated to yield a hit-rate (%). The common spatial pattern (CSP; Ramoser et al., 2000) was applied to these training data and 10 CSP spatial filters that best discriminated the two conditions were selected. These spatial filters projected each training trial into a new domain; finally, the variances of projected signals were used as features. A classifier was generated by Fisher linear discriminant analysis (FLDA), which constructed classification lines in the feature domain between two MI conditions. This classifier was applied to testing data and yielded classification accuracy. Using the same procedure, 120 iterations were performed, thereby yielding 120 estimates of accuracy. The mean of these estimates was used as performance in the given window. As the window was sliding, the best performance time interval for each subject was determined and the best classification accuracy was used as the subject’s MI accuracy. The time interval yielding the best results for each experiment is presented in **Table 1**.

**Table 1:** Experimental information.

Contents	A	B	C	D	E	F	G	H	I	J
**MEG**
Number of channels	152	152	152	152	152	152	152	152	152	152
Sampling rate (Hz)	500	512	512	512	512	512	512	500	500	500
**EEG**
Number of channels	19	19	19	19	19	19	19	19	19	19
Sampling rate (Hz)	512	512	512	512	512	512	512	500	500	500
Start time of 2 s time window for best accuracy (s)	0.4	0.1	0.8	1	0.2	0.5	0.9	0.6	0.6	0.1

### RESTING STATE MEG ANALYSIS

Before or during MEG recording, bad channels showing abnormal behavior were checked carefully; 10 among 152 channels were declared as bad channels and excluded from the analysis. MEG was bandpass-filtered with frequencies between 1 and 100 Hz. Frequency powers were calculated through EEGLAB library (Delorme and Makeig, 2004). We adopted four spectral band ranges: theta (4–8 Hz); alpha (8–13 Hz); beta (13–30 Hz), and gamma (30–70 Hz). The powers of these frequency intervals were summed and normalized by a total frequency power of 4–70 Hz and were divided by the total power over channels. This facilitated our investigation across different channels and subjects without specific channel or band biases. We defined this value as a relative power level (RPL) that was used primarily throughout this work.

## RESULTS

### MI PERFORMANCE

**FIGURE 2:**
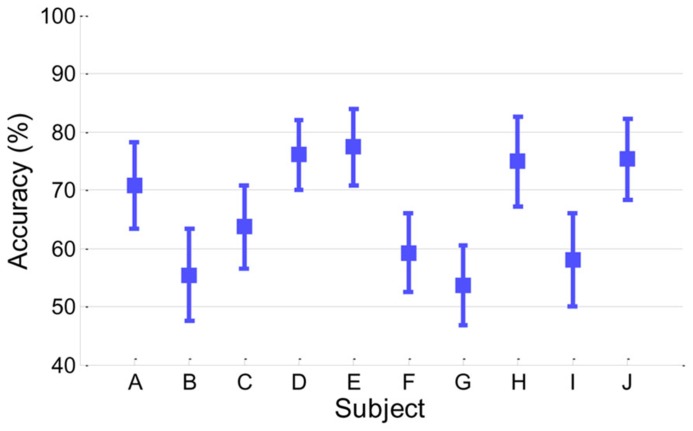
**Classification accuracy in EEG.** Accuracy of each subject is presented with its standard deviation.

**Figure 2** presents classification accuracy estimated by the conventional cross-validation method described in Section “Classification Accuracy From EEG.” Looking at the performance behaviors, five subjects (A, D, E, H, and J) showed reasonably moderate performance (accuracy > 70%) while the other five subjects (B, C, F, G, and I) yielded around chance level (50%) or slightly higher accuracy.

### SPATIAL DISTRIBUTION OF GAMMA ACTIVITY

**FIGURE 3:**
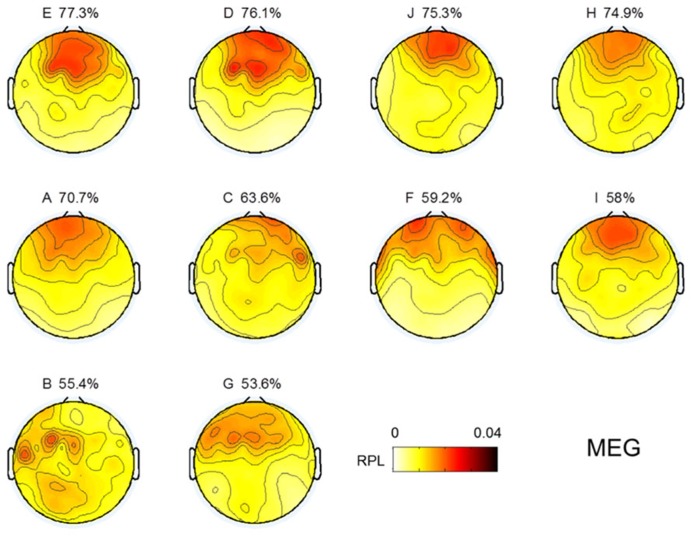
**Topographic images for MEG gamma.** Images are sorted in descending order of MI performance with EEG.

Topographical distributions for gamma RPL over all experiments are illustrated in **Figure 3** under the fixed color bar scale. It is notable that most experiments yielding relatively better classification accuracy had higher levels of gamma in the frontal area. Subject F also seemed to show a high gamma power level in the frontal area; however, high RPL distribution was spread out in the temporal area, and thus was not focused on the frontal mid-line.

### REGION ANALYSIS

**FIGURE 4:**
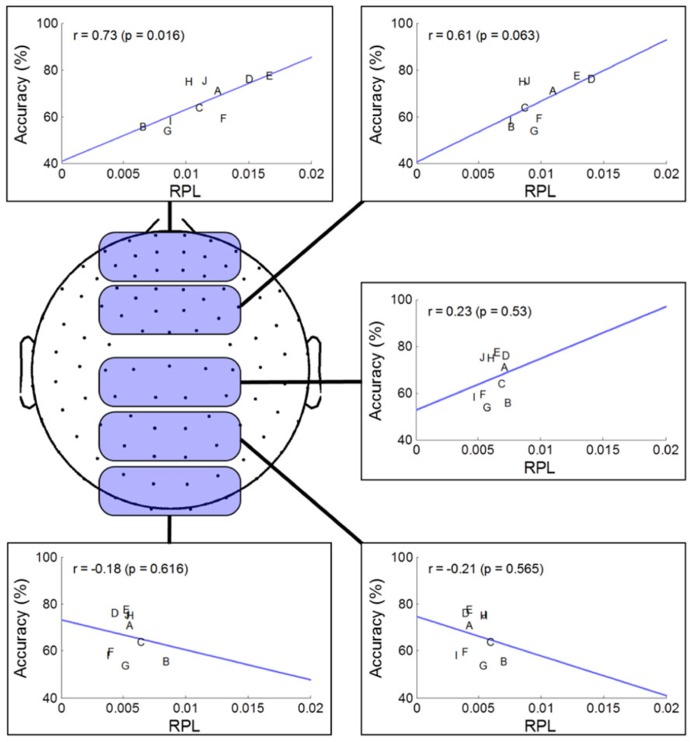
**Regional analysis of MEG gamma.** The selected channels in each region are marked as shaded round squares and their mean gamma RPL is plotted against MI accuracy. The direction of the fitted line and the correlation coefficient changed from the prefrontal to occipital areas.

To investigate spatial gamma effects on MI accuracy, we separated sensor areas into five regions along the midline from the frontal to occipital areas. Through the linear fitting method and Pearson correlation analysis, we observed how averaged resting state gamma levels in each region and MI performance were correlated (**Figure 4**). For statistical validation, we used student *t*-tests for each comparison. In the prefrontal area, we found a highly positive correlation (*r* = 0.73), indicating that gamma level was related strongly to MI performance. Such a positive correlation continued until the frontal area, and then switched to a negative correlation after the central area, where it peaked in the occipital region (*r* = –0.21). Thus, we inferred that frontal gamma may play an important role in MI; meanwhile, the central area was the border at which the relationship changed from a positive to a negative influence on MI performance. Finally, the negative role of gamma activity was maximized in the parietal area. However, looking at the significance level, the negative correlation after the central area was not as high as that of the prefrontal (*p* = 0.02) or frontal areas (*p* = 0.06).

### CORRELATION ANALYSES OF FOUR SPECTRAL BANDS

In the previous section, we found that resting frontal gamma may play a critical role with respect to the capacity for MI, while resting-state gamma power in the centro-occipital region may have a negative association. Such a pattern may affect other spectral rhythms in order to achieve an overall mental state suitable for MI. In this section, frequency band powers other than gamma were investigated using RPL, and these were compared with respect to classification accuracy. For each channel, we conducted Pearson correlation analyses to achieve correlation values and statistical *p*-values from the student *t*-test. Next, a statistical *p*-value topographical distribution for each subject was FDR (false discovery rate)-corrected over all channels for multiple comparison analysis (Benjamini and Hochberg, 1995; Genovese et al., 2002).

**FIGURE 5:**
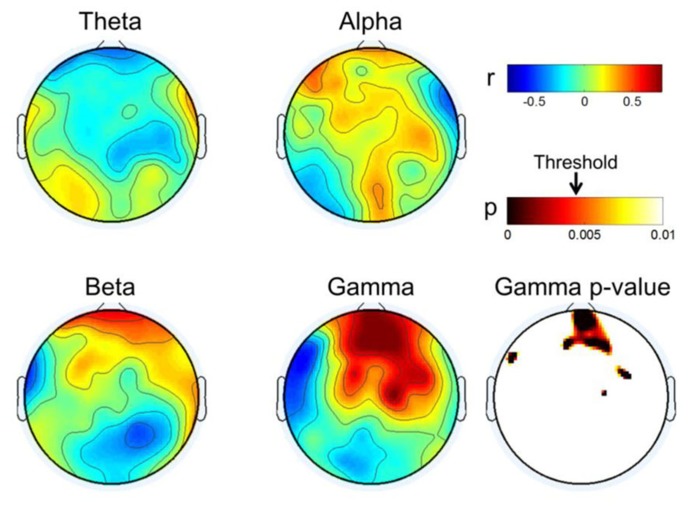
**Topographical plot of correlation distribution between MI performance with EEG and MEG band power.** MEG Gamma *p*-value is shown in the right-bottom; other bands were not statistically significant with FDR-correction threshold (*q* = 0.1). The significant threshold for *p*-values is shown with an arrow in the *p*-value color bar.

**Figure 5** describes the results of correlation analyses for four bands at rest; the findings are summarized as follows:

• Gamma showed positive correlations near the front of the head and those increased in the frontal mid-line area, while other areas showed negative correlations. We inferred from this observation that the frontal midline may be an important point of origin for imagination processing in motor function.
• Beta had a pattern similar to that of gamma; however, it was not focused to the same degree as gamma. Rather, its pattern spread out to cover broad areas.
• Theta showed the reverse pattern to beta and gamma. Correlations near the prefrontal area were negative; theta became weakly positive from the frontal to occipital areas.
• Alpha is known to be the most critical factor in the MI process (Blankertz et al., 2010), especially near the somatic-motor area. This was also observed in **Figure 5** (Alpha). The correlation analysis revealed that alpha had positive correlations over all areas from the front to the back of the head.

## DISCUSSION

### MI PERFORMANCE IN MEG

**FIGURE 6:**
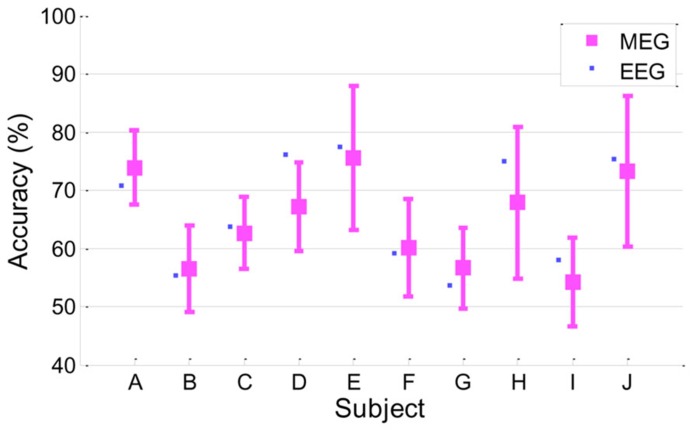
**Classification accuracy in MEG.** MEG accuracy of each subject is presented as a mean with a standard deviation, and for comparison, EEG accuracy is plotted as a small filled square (*r* = 0.91, *p* < 0.0005).

In this work, MI performance was evaluated by EEG because EEG is used more commonly than MEG in the BCI system. However, it is understood that, in general, the MEG signal seems to have a relatively higher signal-to-noise ratio than EEG; thus, it would be quite interesting to see if MEG yields better results than EEG in MI BCI. We investigated MI performance with MEG briefly in a manner similar to EEG. Classification accuracy was estimated through cross-validation as described in Section “Classification Accuracy From EEG.” The same temporal/spectral filtering processing as those in EEG and further channel selection were applied. Next, the best classification accuracy of MEG was chosen. As a result, MEG yielded 64.8 ± 7.9 in classification accuracy, which is slightly inferior to and almost comparable to EEG performance (66.4 ± 9.4). Detailed performances for each subject are depicted in **Figure 6**. Both MEG and EEG performance distributions are correlated strongly (*r* = 0.91, *p* < 0.0005). Even though MEG performance seemed almost comparable to EEG performance in this work, there are still many factors affecting classification performance, such as preprocessing, filtering, feature extractions, and classification. Thus, a solid comparison between MEG and EEG performance is not easy. In addition, we found that the following issues should be considered in MEG processing. First, as reported in Dornhege et al. (2007), a large number of MEG channels may yield over-fitting and it may fail to find reasonable spatial filters. In our brief investigation, we observed that the whole channels (*N* = 152) yielded quite inferior performance (56.7 ± 7.8) to a far smaller number of selected channels (N = 20) when only CSP was applied. Second, compared to EEG electrodes attached on the head, the location of MEG sensors is more likely to move due to head movements of subjects during long experiments. There are reports that location mismatch may also influence classification accuracy (Mellinger et al., 2007; Park et al., 2013). According to Park et al.’s (2013) report, the location change of channels degraded classification accuracy and phase-based features (for example, phase locking value) are more stable than power-based features. Mellinger et al. (2007) recommended using Laplacian spatial filtering rather than more sophisticated methods, such as independent component analysis (ICA), CSP and beamforming (Ahn et al., 2012), where accurate correction of channel location is not guaranteed. In our investigation, we also obtained better accuracy in some subjects by using a time-averaging feature without CSP filtering and rejecting bad trials after examination. In addition, Dornhege et al. (2007) demonstrated that CSP and ICA in MEG could work more poorly than the approach without spatial filtering, while those approaches could improve performance in EEG. In summary, spatial filtering and channel mismatch may be critical factors in MEG processing.

### EEG GAMMA IN RESTING STATE

**FIGURE 7:**
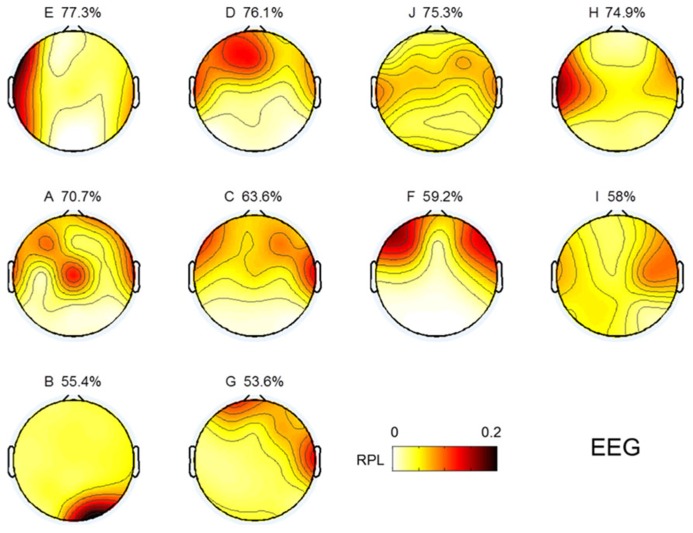
**Topographic images for resting state gamma in EEG**. Images are sorted in descending order of MI performance with EEG.

EEG gamma activity in the resting state was investigated in the same manner as MEG, as shown in **Figure 3**. The topographical images of EEG gamma activity for all subjects are illustrated in **Figure 7**. Due to the small number of EEG channels (*N* = 19), spatial resolution of the EEG image is considerably lower than that of MEG. Unlike MEG gamma in **Figure 3**, any noticeable tendency and statistically significant correlation between resting state gamma and MI performance were not observed in the frontal or centro-occipital areas (*p* > 0.1) in EEG. This result shows that MEG has an advantage over EEG in the investigation of gamma activity. However, this may be due to the specificity of our analysis or the equipment we used here (19 EEG channels).

### MEG GAMMA AND BCI PERFORMANCE

**FIGURE 8:**
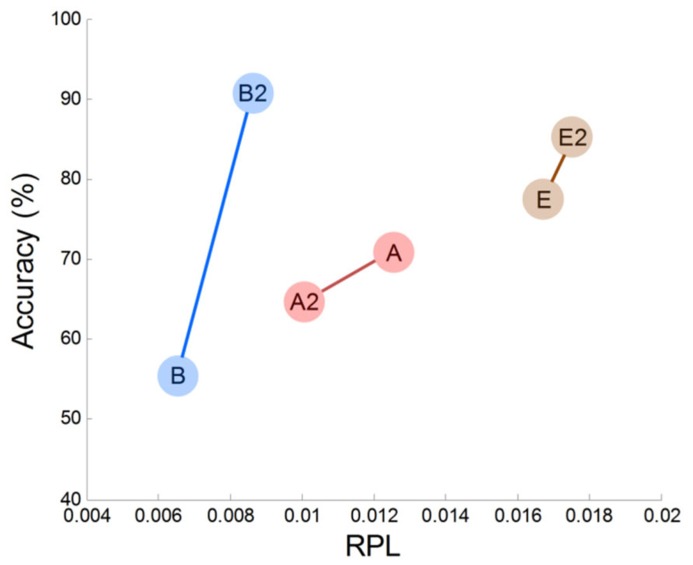
**Prefrontal MEG gamma and accuracy variation in two sessions**.

In this work, we observed empirical evidence that MEG gamma correlates with MI performance. Prefrontal gamma seems to have a strong association with the MI process (at least in our study), but this may be the result of an indirect association. Specifically, we observed that prefrontal gamma yielded highly significant results (*r* = 0.73, *p* = 0.02). Thus, we may infer that frontal gamma is the target neurophysiological factor to differentiate between good and poor MI task performers. However, there are some issues that must be considered further. The first is that frontal gamma may be contaminated by facial electromyogram (EMG) noise. This EMG artifact may yield a substantial effect in our analysis and may be associated with notable patterns in gamma rhythm. To examine such possible EMG effects, we evaluated the correlations of several channels (Fp1, Fp2, F3, F4, F7, F8, Fz) in the frontal area against classification accuracy. We observed that there were no significantly correlated channels; thus, we are convinced that there was no substantial EMG effect in our MEG analysis. The second consideration is that some subjects may not be in a good mental state to conduct imagination. If this is the case, a subject who demonstrates poor accuracy at one time may perform well on another occasion. Because there are reports that attention is related to human performance (Pashler et al., 2001; Liu et al., 2005; Jensen et al., 2007; Lakey et al., 2011; Hammer et al., 2012), the second consideration is well worth investigation. To this end, we compared two different sessions for three subjects who had multi-session data. In **Figure 8**, we plotted frontal gamma RPLs for the first (A, B, C are the same as in **Figure 4**) and second session data (A2, B2 and C2) against BCI performance, and a positive slope over sessions was observed in all three subjects (**Figure 6**). We inferred from this result that high prefrontal gamma during the resting state is likely to yield good classification accuracy. Even though other area channels (frontal, central, parietal and occipital) were investigated similarly, notable patterns such as the positive correlation in the prefrontal area channels were absent. This is an interesting avenue to pursue in future investigations with more datasets.

In addition to our preliminary within-subjects results (**Figure 6**), there are other studies that have demonstrated the importance of prefrontal gamma. Halder et al. (2011) reported high correlations (*r* = 0.72) from fMRI comparisons between MI performance and voxel activations in the prefrontal area. Hammer et al. (2012) concluded that attention resources support BCI performance; importantly, they showed that the ability to concentrate on a task accounted for 19% of the variance in BCI performance. Generally, a high frequency wave like gamma in the prefrontal cortex is interpreted as reflecting attention and memory processes (Jensen et al., 2007; Benchenane et al., 2011). Therefore, one can interpret that the high prefrontal gamma represents considerable activation and synchronization of neurons, and that this facilitates the imagination process for motor function.

Interestingly, the gamma correlations in this study were as high as 0.80 (**Figure 5**), while other bands showed correlations of approximately 0.50 to 0.60. This is a very large range and suggests that frontal gamma may be more important than other bands, considering similar studies showing correlations of *r* < 0.40 across subjects (Ahn et al., 2013) and *r* < 0.10 within subjects (Grosse-Wentrup et al., 2011). We believe that MEG, rather than EEG, may better detect high frequency information, thereby resulting in higher correlations. Although we did not include the results of EEGs recorded during the resting state, it was difficult to see a clear relationship with respect to gamma. Therefore, we may use the advantages of MEG over EEG, and prescreen subjects’ potential performance with MEG before a user begins to play with EEG-based BCI. EEG-based BCI will probably remain the most popular because of its advantages of portability, low cost and simple operation. MEG is large and expensive, and thus it is unrealistic to use for the BCI system. Rather, it is more likely that MEG will contribute to the pre-screening or diagnosis of a user’s traits and mental state.

As does the indirect association in the frontal area, we should understand the direct association with MI-related areas. It is known that larger alpha yields stronger event-related desynchronization, which is used as a most informative feature. This tendency was reported not only in MI (Blankertz et al., 2010; Ahn et al., 2013), but also in memory (Vogt et al., 1998; Klimesch et al., 1999) and visual (Salenius et al., 1995; Doppelmayr et al., 1998) processing. To process a task, it is considered that neurons firing in certain cortical areas are synchronized (Jensen et al., 2007) and that this synchrony produces high frequency oscillations such as gamma. Thus, we may pose the hypothesis that the existing alpha power before MI may shift to rather high frequencies thereafter through neuronal communication in MI-related areas. Thus, low levels of gamma could reflect an idling state in that area, while it is producing high alpha rhythms. In this context, we expected that the centro-parietal gamma rhythm at rest might be correlated negatively with classification accuracy. However, we observed a weak (non-significant) negative correlation with MI performance in the centro-occipital regions (**Figure 5**). Therefore, it was difficult to support our hypothesis, but it is still worth continuing this investigation with more and better data that have good quality high frequency information. We believe that an invasive approach like ECoG may have significant potential in this type of study.

## CONCLUSION

This study employed ten subjects to provide empirical evidence for the importance of gamma in MI accuracy. With simultaneous MEG/EEG data on MI and resting states before and after MI, we found that performance was correlated positively with gamma activity in the prefrontal area. This indicates that the way in which the gamma rhythm is generated in the prefrontal area is of great importance in MI processing. In conclusion, high prefrontal gamma – possibly related to concentration level – represents a good mental state for reaching acceptable performances in MI BCI. This finding will facilitate the development of more advanced BCI designs that reflect users’ mental states in the system itself.

## Conflict of Interest Statement

The authors declare that the research was conducted in the absence of any commercial or financial relationships that could be construed as a potential conflict of interest.
